# Leukocyte telomere length variation due to DNA extraction method

**DOI:** 10.1186/1756-0500-7-877

**Published:** 2014-12-04

**Authors:** Joshua Denham, Francine Z Marques, Fadi J Charchar

**Affiliations:** Federation University Australia, Ballarat, Victoria Australia

**Keywords:** Telomeres, Leukocyte, T/S ratio, qPCR, DNA extraction, High salt method

## Abstract

**Background:**

Telomere length is indicative of biological age. Shorter telomeres have been associated with several disease and health states. There are inconsistencies throughout the literature amongst relative telomere length measured by quantitative PCR (qPCR) and different extraction methods or kits used. We quantified whole-blood leukocyte telomere length using the telomere to single copy gene (T/S) ratio by qPCR in 20 young (18-25 yrs) men after extracting DNA using three common extraction methods: Lahiri and Nurnberger (high salt) method, PureLink Genomic DNA Mini kit (Life Technologies) and QiaAmp DNA Mini kit (Qiagen). Telomere length differences of DNA extracted from the three extraction methods was assessed by one-way analysis of variance (ANOVA).

**Results:**

DNA purity differed between extraction methods used (*P* = 0.01). Telomere length was impacted by the DNA extraction method used (*P* = 0.01). Telomeres extracted using the Lahiri and Nurnberger method (mean T/S ratio: 2.43, range: 1.57 – 3.02) and PureLink Genomic DNA Mini Kit (mean T/S ratio: 2.57, range: 2.24 – 2.80) did not differ (*P* = 0.13). Likewise, QiaAmp and Purelink-extracted telomeres were not statistically different (*P* = 0.14). The Lahiri-extracted telomeres, however, were significantly shorter than those extracted using the QiaAmp DNA Mini Kit (mean T/S ratio: 2.71, range: 2.32 – 3.02; *P* = 0.003). DNA purity was associated with telomere length.

**Conclusion:**

There are discrepancies between the length of leukocyte telomeres extracted from the same individuals according to the DNA extraction method used. DNA purity could be responsible for the discrepancy in telomere length but this will require validation studies. We recommend using the same DNA extraction kit when quantifying leukocyte telomere length by qPCR or when comparing different cohorts to avoid erroneous associations between telomere length and traits of interest.

## Background

In mammalian cells, telomeres are the repetitive sequence (TTAGGG_n_) that prevent end-to-end fusion and maintains chromosomal stability [1]. Leukocytes circulate through many organs and their telomere length correlates to that of other tissues, including fat, muscle, skin and synovial tissue in humans [2–4]. Without the reverse transcriptase enzyme, called telomerase, somatic cell telomeres shorten during mitosis [5]. For this reason and due to ease of accessibility, leukocyte telomere length has become an important biomarker for cellular and biological age. Leukocyte telomere length is shorter in individuals with atherosclerosis [6], type 2 diabetes [7] and cancer [8], and is inversely associated with age and mortality risk [9]. Conversely, lifestyle factors such as a healthy diet, vitamin intake and exercise are associated with longer leukocyte telomeres [10–12].

There are a number of inconsistencies throughout the literature related to telomere length. For example, leukocyte telomere length quantified by real-time quantitative polymerase chain reaction (qPCR) using the telomere to single copy gene ratio (T/S ratio), in which the *36B4* gene is commonly used, varies dramatically throughout the literature and associations between telomere lengths and diseases are sometimes equivocal [13]. The discrepancies could potentially be partially explained by the quantification of telomere lengths after extraction by different DNA extraction methods. We hypothesized that the DNA extraction method used to harvest DNA from leukocytes would impact their telomere length (T/S ratio) quantified by qPCR. Recently, it was demonstrated in a cohort of cancer patients and healthy individuals that telomere length of buffy coat leukocytes was dependent on the DNA extraction method used [13]. Whether telomeres from whole-blood leukocytes are influenced by using other DNA extraction methods remained to be tested. Also, the mechanism by which telomere length varies between extraction methods is not yet understood. Here we compared whole-blood leukocyte telomere length of DNA extracted using three different extraction methods; including two extraction kits that have not been studied in context with telomere length previously.

## Methods

### Sample collection

A resting blood sample was collected from 20 young (18–25 yr) men. Participants self-reported that they were free from any infections and chronic diseases, and were otherwise healthy. Blood was drawn from the antecubital vein into EDTA tubes (BD Vacutainer, Australia) and was immediately stored on ice. DNA was extracted using three individual DNA extraction methods within 12 hours of collection to prevent any *ex vivo* impacts on telomere integrity. DNA yield and purity was checked using a Nanodrop 2000 Spectrophotometer (Thermo Scientific, Australia) before being stored at −20°C. All blood collections were performed in the morning.

All participants gave written informed consent and this study was approved by the Federation University Australia Human Research Ethics Committee.

### DNA extraction

DNA was extracted from whole-blood using the PureLink Genomic DNA Mini Kit (Life Technologies), QiaAmp DNA Mini Kit (Qiagen) and the Lahiri and Nurnberger (high salt) method, a non-commercial, relatively cheap, toxic reagent-free protocol [14]. Reagents for the Lahiri and Nurnberger method were prepared according to guidelines described elsewhere [14]. All DNA extractions were performed according the manufacturers recommendations by one researcher only.

### Telomere assays

Relative telomere length was quantified by qPCR using the T/S ratio [15]. This is a relative measure of telomere length that is strongly correlated to telomere restriction fragments quantified by Southern Blot [15]. The telomere assays were run on the ViiA7 Real Time PCR System (Life Technologies, Australia). Briefly, reactions were run in 384-well plate format with duplicates for samples, an exogenous positive and negative controls. Reaction were comprised of the following constituents: SensiFAST SYBR Lo-ROX master mix (Bioline, Australia), 300 nmol/l of telomere-specific forward and reverse primers, or 300 nmol/l of the single copy gene (*36B4*) forward and 500 nmol/l of the *36B4* reverse primers and 30 ng of DNA to make a total reaction volume of 10 μl. The telomere assay primer-sets have been described elsewhere [16]. A difference of less than one cycle threshold (Ct) between duplicates was required. The telomere assays were repeated on 80% of samples on a separate day to assess the reproducibility of the data.

### Statistics

Kolmogorov-Smirnov and Shapiro-Wilk test were performed and demonstrated the T/S ratios were normally distributed. An ANOVA was performed to determine differences in T/S ratio and DNA purity between the extraction methods studied. Pearson’s Correlations were used to assess linear associations between DNA purity and T/S ratios. Statistical analyses were performed using the IBM SPSS statistics software (version 19) and significance was set at *P* < 0.05.

## Results

DNA extracted from each of the three extraction methods showed significantly different purities (*P* = 0.01, Table 1). Specifically, Lahiri and Nurnberger and Purelink-extracted DNA had lower 260/280 ratios compared with the QiaAmp-extracted DNA (mean ± SEM, 1.7 ± 0.08 vs 1.93 ± 0.01, *P* = 0.003 and 1.78 ± 0.03 vs 1.93 ± 0.01, *P* = 0.048, respectively). There were no statistically significant differences between DNA 260/230 ratios of DNA extracted using the three different extraction methods (*P* > 0.05, Table 1).

**Table 1:** **DNA quality of samples extracted using three extraction methods**

Extraction method	***DNA quality***	
	260/280	260/230
Lahiri and Nunberger	1.7 ± 0.08†	1.45 ± 0.23
Purelink	1.78 ± 0.03‡	1.62 ± 0.1
QiaAmp	1.93 ± 0.01	1.41 ± 0.09

Data are expressed as mean ± SEM from 20 DNA samples extracted using three different extraction methods.

†Lahiri and Nunberger vs QiaAmp (*P* = 0.003).

‡Purelink vs QiaAmp (*P* = 0.048).

**Figure 1:**
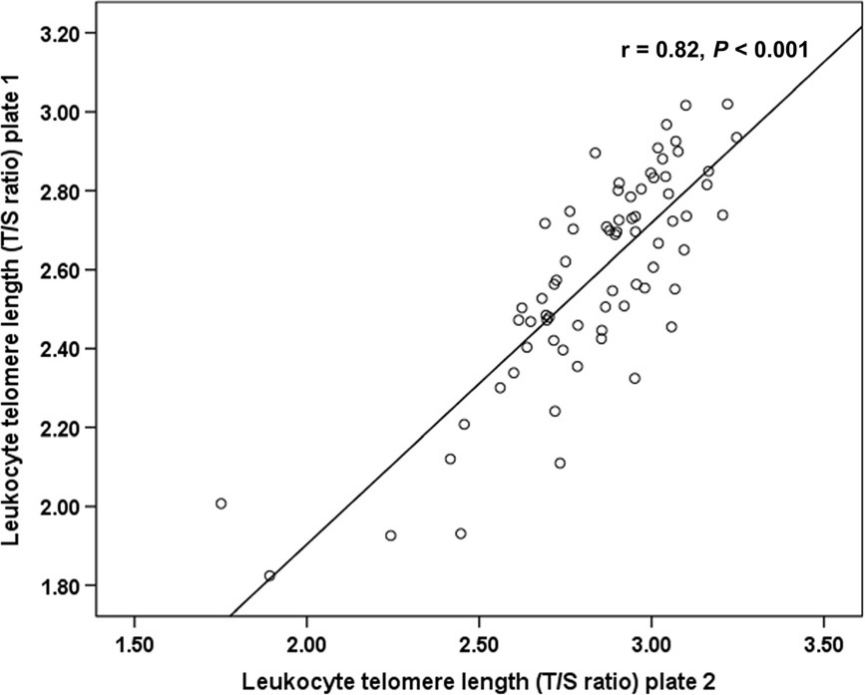
**Linear correlation between telomere assays.** Telomere assays were repeated on 80% of samples on a separate day to assess the reproducibility of the data. Telomere length (T/S ratio) data showed acceptable reproducibility (r = 0.82, *P* < 0.001).

The inter-assay coefficient of variation between independent replicates for the T/S ratio was 9.7% and r-value was 0.82 (*P* < 0.001, Figure 1). The intra-assay coefficient of variation was 1.5% for the telomere primer-set and 0.75% for the 36B4 primer-set, respectively. Therefore, the data showed acceptable reproducibility.

**Figure 2:**
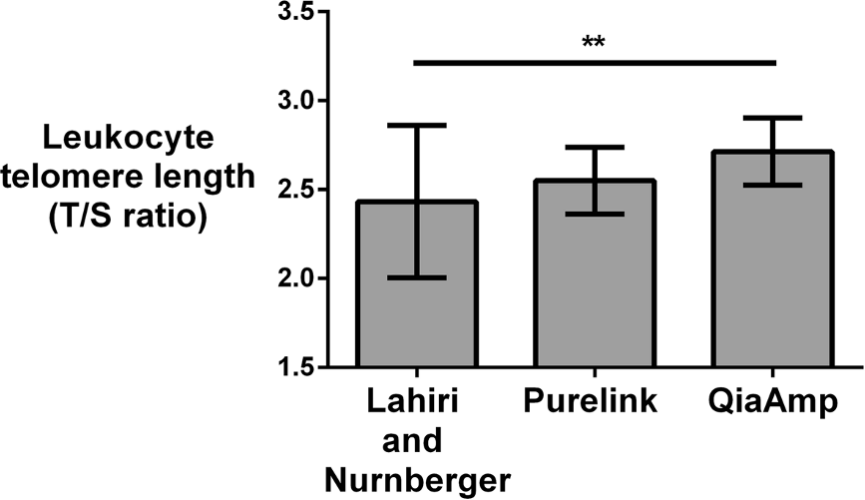
**DNA extraction method impacts leukocyte telomere length (T/S ratio).** Bars are mean telomere length and error bars are standard deviations. DNA was extracted using the Purelink, QiaAmp and Lahiri and Nurnberger methods, respectively. **P = 0.003.

**Figure 3:**
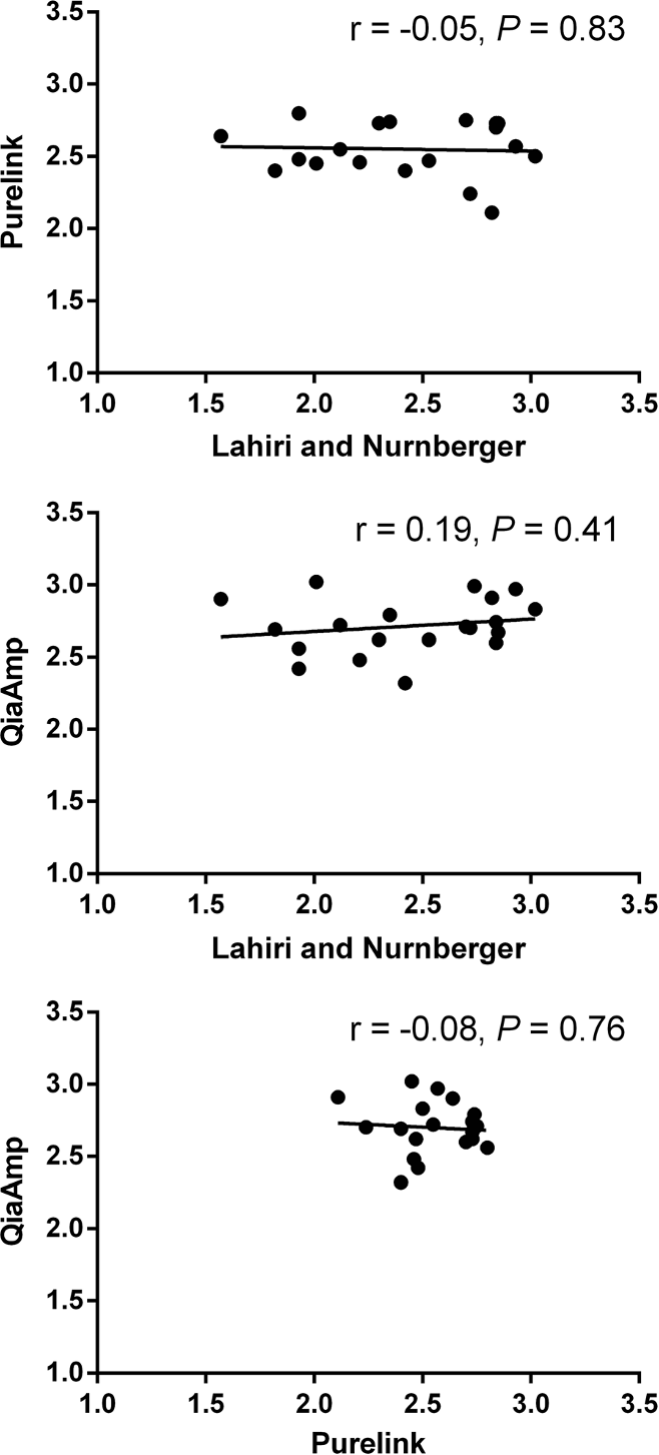
**Correlations between 20 individuals' telomeres using DNA extracted from three different extraction methods.** The x and y axis represents leukocyte telomere length (T/S ratio).

There were significant differences in the average T/S ratio between DNA extracted using the three methods (n = 20, *P* = 0.01). The QiaAmp and Purelink-extracted telomeres were not statistically different (*P* = 0.14). Similarly, telomeres of DNA extracted using the Lahiri and Nurnberger method (T/S ratio: 2.43, range: 1.57 – 3.02) and PureLink Genomic DNA Mini Kit (T/S ratio: 2.57, range: 2.24 – 2.80) did not differ (*P* = 0.13), but Lahiri and Nurnberger extracted telomeres were significantly shorter than those extracted using the QiaAmp DNA Mini Kit (T/S ratio: 2.71, range: 2.32 – 3.02, *P* = 0.003) (Figure 2). There were no statistically significant correlations between the T/S ratios of DNA extracted using the three extraction methods (*P* > 0.05, Figure 3).

**Figure 4:**
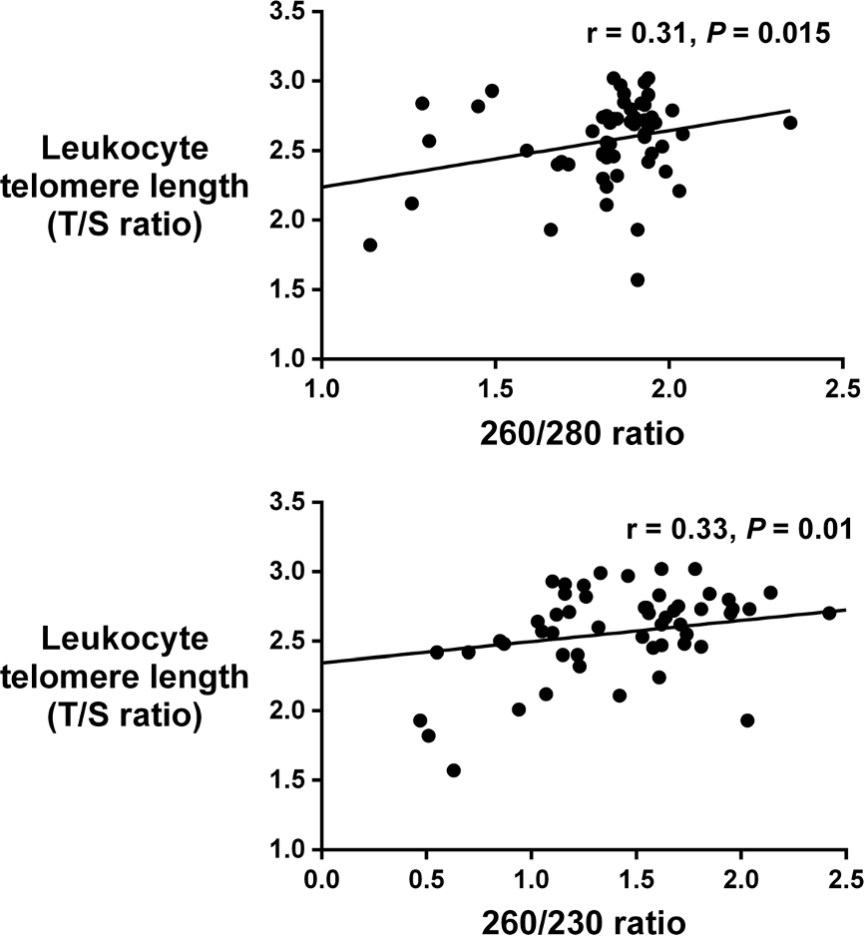
**Correlations between leukocyte telomere length (T/S ratio) and DNA purity (260/280 and 260/230 ratios).**

Finally, there were modest but statistically significant correlations between DNA purity and telomere length (260/280 and T/S ratio: r = 0.31, *P* = 0.015; and 260/230 and T/S ratio: r = 0.33, *P* = 0.01, Figure 4).

## Discussion

Here, we demonstrated leukocyte telomere length quantified by qPCR differs depending on the DNA extraction kit used to isolate the DNA. Furthermore, to our knowledge, we are the first to report telomere length could vary depending on the purity of DNA used for qPCR. Telomere length of DNA extracted from leukocytes using the Lahiri and Nurnberger method were different compared to those extracted using the QiaAmp DNA Mini method. The QiaAmp and Purelink-extracted telomeres were not statistically different. Moreover, telomere length of DNA extracted from the same individual using the three methods were not correlated. Whereas DNA extracted from leukocytes using the Lahiri and Nurnberger method demonstrated the largest variation in telomere length and the shortest average telomere length, the PureLink Genomic DNA Mini method had the least variation in telomere length and the QiaAmp DNA Mini method produced the longest telomeres. There was no statistical significant difference in the length of telomeres extracted using the Lahiri and Nurnberger and PureLink Genomic DNA Mini methods. Additionally, there was no statistically significant difference between telomeres extracted using the QiaAmp and Purelink methods.

Our findings extend previous data on relative telomere length differences between the QiaAmp DNA Mini method and other DNA extraction methods, specifically, the Gentra PureGene Blood Kit and a phenol/chloroform DNA extraction methods (both from Qiagen) [13]. Consistent with a previous report [13], the two column-based extraction methods used in our study, the Purelink and QiaAmp protocols, yielded the smallest range of telomere lengths; the Lahiri and Nurnberger method had the largest telomere length range. Conversely, our data show column-based extraction methods produce longer telomeres compared to a high salt protocol. Particularly relevant is the Lahiri and Nurnberger method, which is a cheap and commonly used method before kits started to be utilized. In addition to extending previous findings [13], our data demonstrate other DNA extraction methods produce different leukocyte telomere lengths in a relatively modest number of participants.

Our novel finding that DNA purity (260/280 and 260/230 ratios) is correlated to telomere length suggests chemical contamination during DNA extraction could influence the qPCR reaction and telomere length. Interestingly, the QiaAmp-extracted DNA had the longest telomeres and the highest 260/280 ratio and the Lahiri and Nurnberger-extracted DNA had the shortest telomeres and lowest 260/280 ratio. The Lahiri and Nurnberger method contains high concentrations of salt which could contaminate extracted DNA. Certainly, additional studies will be required to confirm that differences in telomere length from DNA extracted using different extraction methods is due to DNA purity. We would, however, recommend caution when quantifying and comparing telomeres from DNA with different purities.

The leukocyte telomere length discrepancies observed in the present study could also be a result of telomere damage during the extraction process. The different reagents used in the extraction methods may affect telomere integrity during extraction. For example, both column-based extraction kits (QiaAmp and Purelink DNA Mini) include proteinase K and digestion buffers which effectively lyse proteins and inactivates nucleases. The Lahiri and Nurnberger (high salt) method [14], however, uses non-toxic reagents and a non-ionic detergent, octylphenoxypolyethoxyethanol (Nonidet P-40, Signma-Aldrich), and unlike the column-based kits, may cause residual reagents in isolated DNA during the extraction procedure. This would lead to a decreased DNA purity which might inevitably affect telomere length assessed by qPCR. Shearing of DNA and low molecular weight may also explain differences in telomere length amongst different extraction methods [13]. Both the QiaAmp and Purelink DNA Mini kits isolate DNA 20–50 kb in length. Alternatively, telomere structure may influence telomere length measured by qPCR. Telomeres can form quadruplex structures [17] and telomeric DNA is vulnerable to oxidative damage [18], which may be influenced by some reagents or high concentrations of Na^+^/K^+^ [19, 20]. This may inevitably contribute to altered telomere amplification during qPCR and consequently telomere length. Epigenetic modifications, such as histone acetylation and DNA methylation, are abundant at telomeres and sub-telomeric regions [21], and could also modify the DNA amplification during qPCR. It has been suggested that telomere length differences arising from different DNA extraction methods used are likely to be related to DNA quality and not qPCR amplification effects [13], yet this was not directly analysed. We, however, have demonstrated DNA purity correlates to leukocyte telomere length in healthy young men.

Our results have implications to already published and future research. Any investigations involving telomere length quantification should be required to extract DNA using the same method in order to make justified conclusions regarding the phenotypic association with telomere length. This may be problematic for meta-analyses and large studies that are analyzing a phenotype in context with telomere dynamics, as they involve large data-sets from multiple laboratories, yet the utilization of different extraction methods could yield misleading results. Telomere length has been identified as a clinical relevant prognostic biomarker of disease and disease risk [22–25], and according to our data and that of others [13], the use of different extraction methods across multiple time-points should be avoided. Moreover, as DNA purity could influence telomere length, it could be advantageous to control for DNA purity in statistical analyses when comparing telomere length.

A limitation of our study is that we quantified leukocyte telomere length of 20 young men, a relatively modest number. We have, however, clearly shown that even in a small cohort of young men leukocyte telomere length is impacted by the DNA extraction method used.

Future work should aim at replicating our findings and previous published data [13] suggesting DNA extraction methods influence leukocyte telomere length quantified by qPCR, to confirm results and identify explanations for the discrepancies. The investigation into the effect of other commercial and non-commercial DNA kits on leukocyte and additional cell-type telomere lengths is also warranted. Establishing the cause of the different telomere lengths from different extraction kits is also required. Lastly, it would be beneficial to validate which DNA extraction method is the most valid estimate of telomere length quantified by other methods such as Southern Blot or quantitative fluorescent in situ hybridization (Q-FISH). Interestingly, QiaAmp-extracted leukocyte telomeres were, on average, shorter compared to telomeres extracted by both phenol/chloroform and PureGene protocols when quantified by qPCR but were similar to phenol/chloroform-extracted telomeres when quantified by Southern Blot [13].

## Conclusions

By comparing the length of telomeres extracted from whole-blood leukocytes using three different extraction methods, we have demonstrated that the method of DNA extraction impacts leukocyte telomere length possibly by yielding different DNA purity. We recommend the adherence to a single type of DNA extraction method when performing comparative and prospective telomere length studies in context with health and disease traits.

## Abbreviations

qPCR: Quantitative polymerase chain reaction
T/S ratio: Telomere to single copy gene
LSD: Least significant difference
Ct: Cycle threshold.
